# Correlation between hormone receptor status and depressive symptoms in patients with metastatic breast cancer

**DOI:** 10.18632/oncotarget.15037

**Published:** 2017-02-02

**Authors:** Xiangyu Guo, Junnan Xu, E Ying, Zhifu Yu, Tao Sun

**Affiliations:** ^1^ Department of Internal Medicine 1, Cancer Hospital of China Medical University, Liaoning Cancer Hospital & Institute, Key Laboratory of Liaoning Breast Cancer Research, Shenyang, P. R. China; ^2^ Department of Epidemiology, Cancer Hospital of China Medical University, Liaoning Cancer Hospital & Institute, Shenyang, P. R. China

**Keywords:** breast cancer, hormone receptor, depression, fluoxetine, SDS

## Abstract

Individual differences in depressive symptoms correlate with morbidity and outcomes in breast cancer patients. We evaluated the effect of hormone receptor (HR) status on depressive symptoms in 176 women with metastatic breast cancer at diagnosis. To assess depression, the women completed Self-Rating Depression Scale (SDS) questionnaires at baseline examination (T1), after 4 chemotherapy cycles (T2) and after 6 months (T3). At baseline examination, 45/176 (25.6%) patients were found to be at high or medium risk for depression (SDS score ≥0.6). Among these, depression was both prevalent in HR-positive patients and in HR-negative patients (64.4% versus 51.4%, *P* = 0.001). In multivariate model, HR positivity and higher depression risk were associated with poorer overall survival (25.0 months versus 32.0 months, *P* < 0.05). Patients at high/medium risk of depression were treated with the antidepressant agent fluoxetine (N = 23) or no drug (N = 22). SDS scores in patients treated with fluoxetine were lower after 4 chemotherapy cycles and after 6 months than in the control group (mean scores: T2, 0.61 versus 0.67, *P* = 0.001; T3, 0.56 versus 0.65, *P* < 0.001). No difference on SDS scores was found between patients with positive or negative HR status during fluoxetine treatment. These findings suggest hormone receptor status is associated with depressive symptoms in patients with metastatic breast cancer. Fluoxetine relieves depressive symptoms in these patients, regardless of hormone receptor status.

## INTRODUCTION

Breast cancer is the most frequently diagnosed cancer in women and the leading cause of cancer-related mortality among them [1]. Approximately 60-75% of patients with breast cancer are hormone receptor (HR)-positive at diagnosis, where HR refers to estrogen receptor (ER), progesterone receptor (PR), or both. ER and/or PR positivity typically carry a favorable outcome and are prognostic predictors of response to endocrine therapy for breast cancer [2]. Implemented as adjuvant or for metastasis treatment, endocrine therapies generally improve progression-free survival and overall survival in patients with HR-positive breast cancer [3].

A recent study demonstrated that patients with breast cancer have considerable distress and depressive symptoms [4]. Unipolar depression, the most common psychiatric disorder, is characterized by extremely low mood and loss of interest in activities of daily life and is associated with biochemical alterations including decreased 5-HT_2b_ receptor levels and increased cortisol and 5-HT levels [5–6]. Depression is associated with substantial morbidity not only due to its related incapacitating symptoms [7], but also because it is often related to chronic physical disease [8–9]. A recent meta-analysis suggested that depression presents a small, marginally significant association with subsequent overall cancer risk [10]. In this regard, hormone-related cancers are of particular interest due to the relevance of several endocrine signaling pathways possibly interacting with 5-HT and other signaling pathways [10–11].

Depression in cancer patients is known to be influenced by diagnosis, choice of treatment, and therapy side effects, but is often neglected as a psychological/psychiatric factor that may be important to post-treatment adjustments [12]. It has been shown, for instance, that the level of depression and anxiety increase after diagnosis of breast cancer [13]. Unfortunately, limited clinical information is available for women with metastatic breast cancer regarding risk factors related to depressive symptoms. This is critical since depressed patients tend to refuse and withdraw anti-tumor chemotherapy more often than non-depressed ones [14]. A clear understanding of depression could make it possible to implement strategies to reduce psychological burden and improve survival. This premise has been validated by retrospective and prospective studies dealing in particular with clinical outcomes of patients with depressive symptoms [15–16]. Several reports also indicated that endocrine treatment -particularly tamoxifen- success was strongly related to depressive symptoms in patients with breast cancer [17–18].

Thus, given the high prevalence of depression and its increasingly recognized association with disease progression and survival, understanding the full magnitude of the relation between depression and the pathological characteristics of cancer seems critical. Little is known regarding the association of HR status and depressive symptoms in female patients with metastatic breast cancer. In this study, we aim to examine whether depression is an independent factor for overall survival, as well as the association of hormone receptors with depressive symptoms in patients with metastatic breast cancer.

## RESULTS

### General characteristics

One hundred and seventy-six women patients with breast cancer were enrolled in the present study. As shown in Table 1, the median age was 49.5 years (age range from 28 to 80). All participants presented with metastatic breast cancer: 67 patients had lung metastases, 71 patients had liver metastases, and 114 patients had bone metastases. A majority (143/176) of the patients had multiple metastases (more than two metastatic sites). A total of 176 tumors were available for ER and PR expression analysis, of which 99 (56.3%) were ER-positive and 84 (47.7%) were PR-positive. A total of 154 tumors were available for assessment of human epidermal growth factor receptor 2 (HER-2) analysis, of which 42 (23.9%) were HER-2-positive and 112 (63.6%) were HER-2-negative.

**Table 1:** Clinicopathologic characteristics of the study sample

	All N = 176	Major/medium N = 45	Minor N = 59	Not depressed N = 72
**Age (yrs)**				
**Median [range]**	49.5 [28,80]	51 [30,80]	50 [30,76]	48 [28,75]
**Menopausal status**	N = 166	N = 43	N = 54	N = 69
Premenopausal	50 (28.4)	9 (20.0)	17 (28.8)	24 (33.3)
Postmenopausal	116 (65.9)	34 (75.6)	37 (62.7)	45 (62.5)
**BMI**	N = 175	N = 45	N = 59	N = 71
**Median [range]**	24.1 [16.^7^,^33^.^2^]	24.2 [16.7,31.3]	24.0 [18.8,30.5]	24.1 [17.7,33.2]
**Tumor size (cm)**	N = 102	N = 27	N = 37	N = 38
**Median [range]**	2.35 [0.7-6.0]	2.50 [1.2-5.1]	2.0 [0.8-6.0]	2.75 [0.7-6.0]
**Lymph node Met**				
Negative	36 (20.4)	11 (24.5)	10 (16.9)	15 (20.8)
Positive	114 (64.8)	28 (62.2)	41 (69.5)	45 (62.5)
Unknown	26 (14.8)	6 (13.3)	8 (13.6)	12 (16.7)
**ER**				
Positive	99 (56.3)	29 (64.4)	34 (57.6)	36 (50.0)
Negative	77 (43.7)	16 (35.6)	25 (42.4)	36 (50.0)
**PR**				
Positive	84 (47.7)	25 (55.6)	33 (55.9)	26 (36.1)
Negative	92 (52.3)	20 (44.4)	26 (44.1)	46 (63.9)
**HER-2**				
Positive	42 (23.9)	7 (15.6)	10 (16.9)	25 (34.7)
Negative	112 (63.6)	32 (71.1)	43 (72.9)	37 (51.4)
Unknown	22 (12.5)	6 (13.3)	6 (10.2)	10 (13.9)
**Histologic type**				
Ductal	140 (79.6)	36 (80.0)	46 (78.0)	58 (80.6)
Lobular	12 (6.8)	4 (8.9)	3 (5.1)	5 (6.9)
Others	24 (13.6)	5 (11.1)	10 (16.9)	9 (12.5)
**BDFS**				
<24 months	64 (36.4)	11 (24.4)	20 (33.9)	33 (45.8)
≥24 months	112 (63.6)	34 (75.6)	39 (66.1)	39 (54.2)
**Lung M**				
Positive	67 (38.1)	18 (40.0)	20 (33.9)	29 (40.3)
Negative	109 (61.9)	27 (60.0)	39 (66.1)	43 (59.7)
**Liver M**				
Positive	71 (40.3)	14 (31.1)	27 (45.8)	30 (41.7)
Negative	105 (59.7)	31 (68.9)	32 (54.2)	42 (58.3)
**Bone M**				
Positive	114 (64.8)	26 (57.8)	36 (61.0)	51 (70.8)
Negative	62 (35.2)	19 (42.2)	23 (39.0)	21 (29.2)

### Hormone receptor status is associated with depression

At baseline (T1), and regardless of hormone receptor status, of all eligible patients screened (N = 176) 25.6% (n = 45) were found to be at major/medium depression risk; 33.5% (N = 59) showed minor depression symptoms; and 41% (N = 72) were not depressed. Stratification analyses showed that ER and PR positivity, and HER-2 negativity, were more frequently associated with overall depression risk (Table 1). ER positivity was detected in 64.4% of patients with major/medium depression, in 57.6% of patients with minor depression, and in 50% of patients with no depression. PR positivity was found in 55.6% of patients with major/medium depression, in 55.9% of patients with minor depression, and in 36.1% of patients with no depression. While ER status made no difference in non-depressed patients, most of these patients were PR negative (Table 1). Pooled data revealed that depression (both major and minor) was positively associated with HR status (64.4% versus 51.4% of HR-negative patients, *P* = 0.001).

### Fluoxetine improves depressive symptoms in patients at higher risk for depression

**Figure 1:**
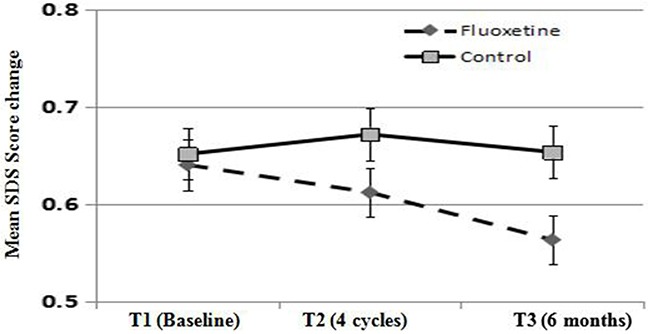
Depression score values at 4 cycles (T2) after chemotherapy and 6 months (T3) after baseline examination (T1) Fluoxetnine indicates fluoxetine treatment group. Control indicates control group. SDS, self-rating depression scale.

A total of 45 patients at higher risk for depression at baseline were randomized to the antidepressant fluoxetine (20 mg; n = 23) or no drug (control group; n = 22). Patients were not allowed to take any other antidepressant drugs or herbal medicines during treatment. As shown in Figure 1, at T1, no significant differences in SDS scores were observed between groups: 0.65 versus 0.64 for fluoxetine and control, respectively (t = 0.629, *P* = 0.533). At T2 and T3, SDS scores decreased significantly in the fluoxetine group compared with the control group (mean scores: T2, 0.61 versus 0.67, t = 3.446, *P* = 0.001; T3, 0.56 versus 0.65, t = 5.041, *P* < 0.001) (Figure 1). Sub-analysis by HR status revealed no significant differences between the HR-positive group and the HR-negative group (HR = 0.95, 95% CI 0.87-1.13, *P* > 0.05).

### Overall survival outcomes

**Figure 2:**
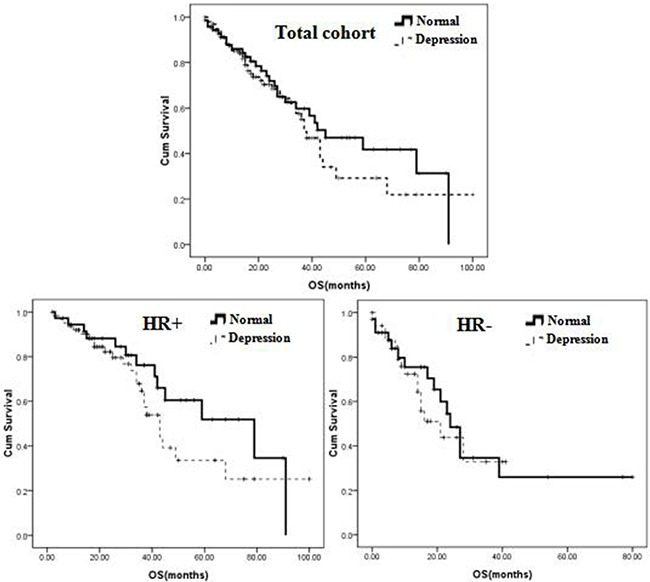
Survival curve showing overall survival for metastatic breast cancer patients with major depression receiving fluoxetine or no drug (control)

The median follow-up time was 32 months (range: 2-89 months). Eighty-five (48.3%) deaths were recorded. No significant differences were detected for median overall survival (OS) between depressed and non-depressed patient groups. As shown in Figure 2, median OS was 18.5 months (1-100 months) in patients with depressive symptoms versus 22.0 months (1-91 months) for those with no depression (*P* = 0.420). A significant difference in OS was observed, however, for patients with HR-positive breast cancer, in which the median OS was 25.0 months for those with depressive symptoms versus 32.0 months for those without depression (*P =* 0.049). In contrast, depression status did not affect OS in HR-negative breast cancer (median OS of 14.0 months in patients with depressive symptoms versus 15.0 months in patients without depression (*P* = 0.219) (Figure 2).

## DISCUSSION

A growing number of studies are addressing the prevalence of depressive symptoms and distress in cancer patients, and their impact on clinical outcomes and quality of life. It is estimated that, similarly to other cancer types, one out of five patients with breast cancer suffer from depression after anti-cancer therapy [19]. In the US, the lifetime prevalence of major depressive disorder is more than 12% in males and 20% in females [20]. Worldwide, major depressive disorder has been estimated to affect 14.6% of people in developed countries and 11.1% of people in less developed countries [21–23]. A study conducted in Germany determined that the levels of depression and anxiety increase after diagnosis of breast cancer in both general and gynecological practices [13].

Early detection and treatment of depression is important to reduce the suffering caused to the patients and increase compliance with anti-tumor therapies [15–16]. Hormone-related cancers include breast cancer, prostate cancer, and endometrial and cervical cancer, and several potential mechanisms through which depression may influence the development of these cancers have been posited. They include a role for sex hormones in both cancer and depression, dysregulation of the hypothalamic-pituitary-adrenal (HPA) axis, increased cortisol response, altered immunological and inflammatory pathways, and inhibition of DNA repair mechanisms [24]. The molecular mechanisms underlying the development of depressive symptoms in patients with breast cancer are not well understood.

Reported risk factors related to depression in patients with breast cancer are loneliness, helplessness, and income or education level, rather than pathological characteristics [25]. Although little is known about whether depressive symptoms are directly related with poor overall survival, a causal link is suggested by the fact that breast cancer patients with depression tend to refuse and/or withdraw potentially effective anti-tumor therapies [15, 26]. Therefore, continuous assessment of depressive symptoms may be critical to improve compliance. Burgess et al. found that depressive symptoms decrease over time after breast cancer diagnosis, while recurrence is associated with a sharp increase in depression and anxiety in a high-risk population [27]. Thus, depressive symptoms combined with low treatment compliance are quite plausibly associated with reduced disease-free survival and overall survival. In our study, we aimed to investigate the relationship between depression and HR status in female patients with metastatic breast cancer. Combined data from ER and PR expression analyses showed that a significantly higher risk for depressive symptoms was present in HR-positive patients. On the other hand, although depression *per se* was not found to affect OS in patients with metastatic breast cancer, a shorter median OS was observed among HR-positive, depressed patients, compared with non-depressed ones (25.0 vs 32.0 months).

Tamoxifen, as an important endocrine agent, has for decades contributed to a robust decrease in breast cancer recurrence and mortality from both primary and metastatic breast cancer; however, tamoxifen causes estrogen insufficiency and depressive symptoms [28–29]. In line with our findings, a retrospective study suggested that chemotherapy and ER-positive status were both associated with an increased risk for developing depression symptoms, independently of tamoxifen exposure [30]. In our cohort, most patients with metastatic breast cancer received several endocrine agents with diverse anti-cancer actions in systemic (adjuvant/ metastasis treatment) therapy sets. Because of this, it was difficult to identify and analyze the differential effects of diverse endocrine agents on major and minor depression, and our study failed to demonstrate a significant association between tamoxifen treatment and depression. Although some studies and several case reports have suggested an association between tamoxifen and depression symptoms, two large, placebo-controlled, randomized studies found no significant difference between tamoxifen and placebo treatment groups on triggering or exacerbating depression [31–32].

Depression is normally associated with various other symptoms such as fatigue, and low quality of life [33–34]. Fluoxetine, as an antidepressant medication for major depression disorder, presents a favorable risk-benefit profile compared with other antidepressant medications such as paroxetine, citalopram, sertraline, and venlafaxine [35]. In this study, fluoxetine proved to be an effective therapy for depression in metastatic breast cancer patients. At T2 and T3, compared with controls, SDS scores decreased significantly in the fluoxetine group (mean scores: T2, 0.61 versus 0.68, *P* = 0.063; T3, 0.51 versus 0.64, *P* = 0.041). Although its effectiveness was modest, perhaps as a result of our limited patient population sample, our results suggest that antidepressant medication may be of benefit during anticancer therapy. Moreover, antidepressants may benefit breast cancer patients regardless of HR status, since no significant difference in the response to fluoxetine was observed between HR-positive and HR-negative patients.

Our study presents some limitations. First, a convenient and inexpensive self-reported questionnaire (SDS) was used for grading patients’ depression. As diagnosis by a mental health professional using standardized diagnostic criteria is a more valid measure of depressive symptoms, the SDS method is a general limitation in studies like ours. Second, HR status, namely ER and PR expression, were detected in primary and metastatic tumor specimens, with a low discordance rate of 6.25% (11/176) between primary and metastatic tumor specimens, and HR status data were included only for the latter in statistical analyses. Third, a placebo was not used in the control group to evaluate the response to fluoxetine, and the study included a small number of patients (45) with major or medium depression. In addition, only 26.1% (6/23) patients completed the six-month fluoxetine treatment. Because major depressive disorder typically manifests chronic and/or recurrent symptoms, and tumor recurrence most often occurs within 6-12 months after remission, further studies on patient responses to continued treatment of fluoxetine for more than six months should be conducted in a larger sample.

In conclusion, HR positivity was associated with increased risk of depression in patients with metastatic breast cancer. Depression, in turn, was associated with shorter OS in these patients. A six-month course of fluoxetine improved depressive symptoms regardless of HR status. Further studies on the mechanisms underlying the association between depression and hormone imbalance in breast cancer should be conducted in patients with diverse HR statuses.

## MATERIALS AND METHODS

### Study participants

From December 2011 until February 2016, 197 women with metastatic breast cancer from the Cancer Hospital of China Medical University, Liaoning Cancer Hospital & Institutes, were enrolled. This study was approved by the Liaoning Cancer Hospital and Institute ethical committee (approval number: 20150903). Among study participants, 21 patients (3.1%) with missing information were excluded, and finally, 176 patients (medium age, 49.5 years; age range, 20-82 years) were included in this study. Histopathological tumor types were invasive ductal type (N = 140), invasive lobular carcinoma (N = 12), and other specified carcinomas (N = 24). Baseline characteristics of the patients are summarized in Table 1.

### Hormone receptor and HER-2 expression analysis

For analysis, HR (ER and PR) and human epidermal growth factor receptor 2 (HER-2) evaluations were performed on surgical specimens and/or core biopsy with immunohistochemistry methods in primary and metastatic tumors. When HER-2 results were equivocal (weakly positive, 2+) additional molecular testing was carried out using fluorescent *in situ* hybridization (FISH). HR heterogeneity can affect precise receptor status determination; discordance rate was 6.25% (11/176) between primary and metastatic tumor specimens. Information derived only from metastatic tumor specimens was used for statistical analyses.

### Study design

Self-Rating Depression Scale questionnaires (SDS) were administered to patients at three time points: at baseline examination, before starting new therapy (T1); after 4 chemotherapy cycles (T2); and after 6 months (T3). All the patients provided written informed consent after the purpose, procedures, risks, and benefits of the study were explained and all their questions were answered.

### Depression assessment

Participants completed the SDS questionnaire at each of the three time points described above. The SDS, a 20-item self-rating scale of cognitive-affective and somatic depressive symptoms, has been used widely in breast cancer patients and is supported by confirmatory factor analysis. The depression index score was calculated as the total score from 20 questions divided by 80 (the maximum possible score). Scores ranging 0-0.49, 0.50-0.59, 0.60-0.69, and 0.70-1.00 indicate no depression, minor depression, medium depression, and major depression, respectively.

### Fluoxetine treatment

Of the 176 patients included in the study, 45 patients (25.6%) were found to be at high or medium risk for depression (SDS score ≥ 0.60). These patients were randomly assigned to treatment or control groups in a 1:1 ratio, and treated, respectively, with the antidepressant agent fluoxetine or with no drug. Twenty-three patients received a daily dose of 20 mg of fluoxetine for at least 6 months.

### Statistical analysis

All statistical data is presented as mean ± SD. Analyses were performed using SPSS 20.0 for Windows (SPSS Inc., Chicago, IL, USA). Survival time was calculated as the time period from the beginning of treatment for metastatic breast cancer until date of death or last follow-up visit recorded. Survival curves were estimated by the Kaplan-Meier method. In Cox proportional hazard models, participants with major, medium and minor depression were compared with participants with no depression. Statistical analysis of the efficacy of fluoxetine on improving depression at different treatment times was performed using independent t-test. Statistical significance was set at *P* < 0.05. All reported *P* values are two-sided.

Provision of study materials or patients: Xiangyu Guo, Junnan Xu, Ying E.

Data analysis and interpretation: Junnan Xu, Xiangyu Guo, Zhifu Yu.

Manuscript writing: All authors.

Final approval of manuscript: All authors.
